# Supplementary material for: Content-rich biological network constructed by mining PubMed abstracts
Source: BMC Bioinformatics. 2004 Oct 8;5:147. doi: 10.1186/1471-2105-5-147 (PMC528731; doi:10.1186/1471-2105-5-147)
Supplement: Additional File 5 — The original Chilibot query results of the term "long-term potentiation (LTP)" and 22 other terms, limiting the latest references analyzed to the years 1990, 1995, 2000, and 2004. [file 1471-2105-5-147-S5.bz2 › chilibotAdditionalFile5/ltp2004/html/ZIF268_SYNAPTOPHYSIN.html]

 


 **ZIF268** and **SYNAPTOPHYSIN** 
  
Found 2 abstracts in PubMed,  **2 abstracts were retrieved and analyzed**.  


---

 Search Google  |
 PDF files only 
|  EDU domain only 

---

**Interactive relationship** (e.g. stimulation, inhibition, etc)

**Parallel relationship** (e.g. studied together, co-existance, homology, etc.)

- PRP resulted in transneuronal changes in the relative distributions of CO,  **Zif268** ,  **synaptophysin** , and GAP 43 in the primary visual cortex.  Ref: 11797318 Trans Am Ophthalmol Soc, 2001
- PURPOSE To determine the effects of panretinal photocoagulation on the levels of cytochrome oxidase,  **Zif268** ,  **synaptophysin** , and growth associated protein 43 in the primary visual cortex of adult monkeys.  Ref: 11797318 Trans Am Ophthalmol Soc, 2001
- At 20 hours, 12 days, 6 months, and 13 months after laser treatment, the visual cortex was assessed histologically for CO and immunohistochemically for  **Zif268** ,  **synaptophysin** , and GAP 43.  Ref: 11797318 Trans Am Ophthalmol Soc, 2001
